# Behavior of colloidal gels made of thermoresponsive anisotropic nanoparticles

**DOI:** 10.1038/s41598-022-16414-w

**Published:** 2022-07-15

**Authors:** Long Yang, Héloïse Thérien-Aubin

**Affiliations:** 1 School of Textile Science and Engineering, Xi’an Polytechnic University, Xi’an, China; 2 Max-Planck Institute for Polymer Research, Mainz, Germany; 3 Department of Chemistry, Memorial University of Newfoundland, St. John’s, Canada

**Keywords:** Chemistry, Materials science, Nanoscience and technology

## Abstract

Amongst colloidal gels, those designed by the assembly of anisotropic colloidal particles tend to form fibrillar gels and are attracting interest as artificial cell growth environments since they have a structure reminiscent of biological extracellular matrices. Their properties can be tuned by controlling the size, shape, and rigidity of the nanoparticles used during their formation. Herein, the relationship between the physical and mechanical properties of the nanocolloidal building blocks and the properties of the resulting gels is investigated. Thermoresponsive particles with different aspect ratios and controlled rigidity were prepared, and the gelation and the properties of the resulting gels were studied. The results show how the aspect ratio and rigidity of polymer colloids tune the properties of the gels. An increase in the aspect ratio of the nanocolloid used led to a sol–gel transition observed at lower particle concentration, but an increase in the rigidity of the nanocolloids delayed the sol–gel transition to higher concentration. However, at a constant concentration, increases in the anisotropy produced gels with higher modulus and lower yield strain. Similarly, an increase in rigidity of the colloids increased the modulus and reduced the yield strain of the resulting gels.

## Introduction

Colloidal gels are transient but arrested networks of colloidal particles^1,2^. The transition between liquid-like colloidal suspensions and the formation of colloidal gels depends on the volume fraction occupied by particles and the range and type of interaction between the particles^3,4^.Colloidal gels can be used in applications such as colloidal crystals^5,6^, energy storage devices^7^, advanced ceramic materials^8^, and biomaterial^9^, due to their unique structure, elasticity, and mechanical stability. Harnessing the interplay between the physico-chemical design of the nanocolloids and the properties of the resulting colloidal gels is consequently increasingly important for manufacturing a vast range of applied materials.

The mechanisms leading to the formation of colloidal gels are complex. When the colloidal particles, used as the building blocks for the formation of the colloidal gel, are simple unfunctionalized rigid spherical particles, the gel formation is predominantly driven by the volume fraction occupied by the particles, and by particle-particle interactions. However, when the colloids are functionalized with polymer chains, the situation is more complex because interdigitation and entanglement between the coronas of adjacent particles affect the particle–particle interaction^10^. Furthermore, when the building blocks are anisotropic and flexible, such as worm-like micelles, the entanglements between the colloids also play a role in the final properties of the colloidal gels^11^.

Understanding and controlling the structure and the mechanical properties of the colloidal gels is crucial to develop materials for specific applications, e.g., photocatalysis^12^, sensors for chemical detection^13,14^, tissue engineering^15^. The colloidal building blocks can be functionalized and are tunable in terms of composition, size, shape, and rigidity. This provides adaptable systems and increases the diversity of colloidal gels and their potential application. For example, fibrilar colloidal gels prepared using anisotropic colloids can lead to the formation of gels showing potential as matrices for cell culture^16–18^. The fibrous structure of those gels is reminiscent of the structure of the natural extracellular matrix^19^. For such application, finely controlling the mechanical properties of the gel is critical because it affects the fate of the cells in the gel and the type of tissues that can be grown in a given gel^20–23^.

The mechanical behavior of colloidal suspensions and gels is influenced by multiple factors, such as size, shape, surface functionalization, and softness of the nanoparticles^24,25^. Soft colloids like microgels are known to interact with each other more strongly than hard colloids and can lead to the formation of stronger colloidal gels^26^. Even in the melt, soft and elastic polymer nanoparticles have been shown to interact more strongly due to the more accessible chain entanglement observed in loosely crosslinked particles^27^. The colloid–colloid interaction is a critical parameter that is also influenced by the surface-functionalization of the colloids. For example, when colloids are functionalized with end-tethered polymer chains, interpenetration and interaction between the grafted polymer layers will dictate the mechanical properties of the resulting colloidal gels^10^. The colloid-colloid interaction is influenced by the core-core interaction leading to the onset of the jamming regime, and the compression and interpenetration of the canopy of end-tethered chains create new pathways of stress dissipation^28^. Moreover, colloids functionalized with limited chain grafting density and same chain length experience a more efficient interpenetration between the brush layer of adjacent colloids leading to stronger and more robust gels^29,30^. In the case of anisotropic colloids, their mechanical properties are similarly influenced by their surface functionalization and the softness of the nanoparticles, but also affected by their aspect ratio and their orientation^31^. Anisotropic colloids have a lower percolation threshold in comparison to their spherical counterpart, and hence fibrillar gels can be formed at low solid content or volume fraction^32^. In this work, we study how the physical properties of anisotropic nanoparticles with the same surface chemistry and chemical composition but having different aspect ratios and rigidity influence the global properties of the colloidal gel, in doing so, developing design rules for the formation of fibrillar gels with well-defined properties.

## Results and discussion

**Figure 1:**
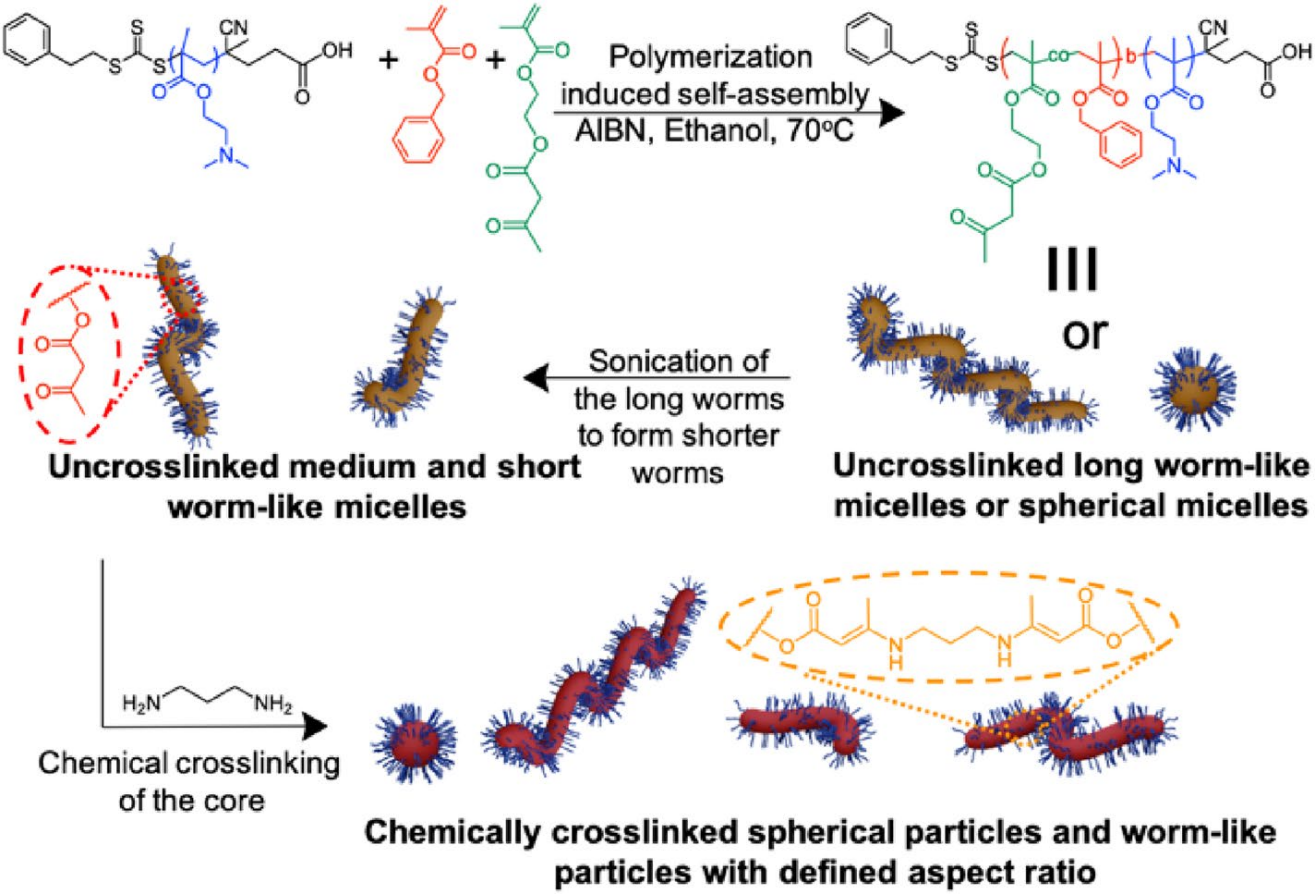
Synthesis of core crosslinked stable block copolymer nanoparticles through polymerization induced self-assembly, sonication and post-polymerization crosslinking. First, a macro-CTA composed of poly(2-(dimethylamino)ethyl methacrylate is extended by the polymerization of a mixture of benzyl methacrylate and 2-(methacryloyloxy)ethyl acetoacetate. Controlling the length of the second block leads to the formation of either spherical micelles or long worm-like micelles. Then, ultrasonication cuts the long worm-like micelles into smaller worm micelles. Finally, the acetoacetate groups present in the core of the micelles are crosslinked by the addition of diaminopropane to yield the chemically crosslinked nanoparticles.

**Figure 2:**
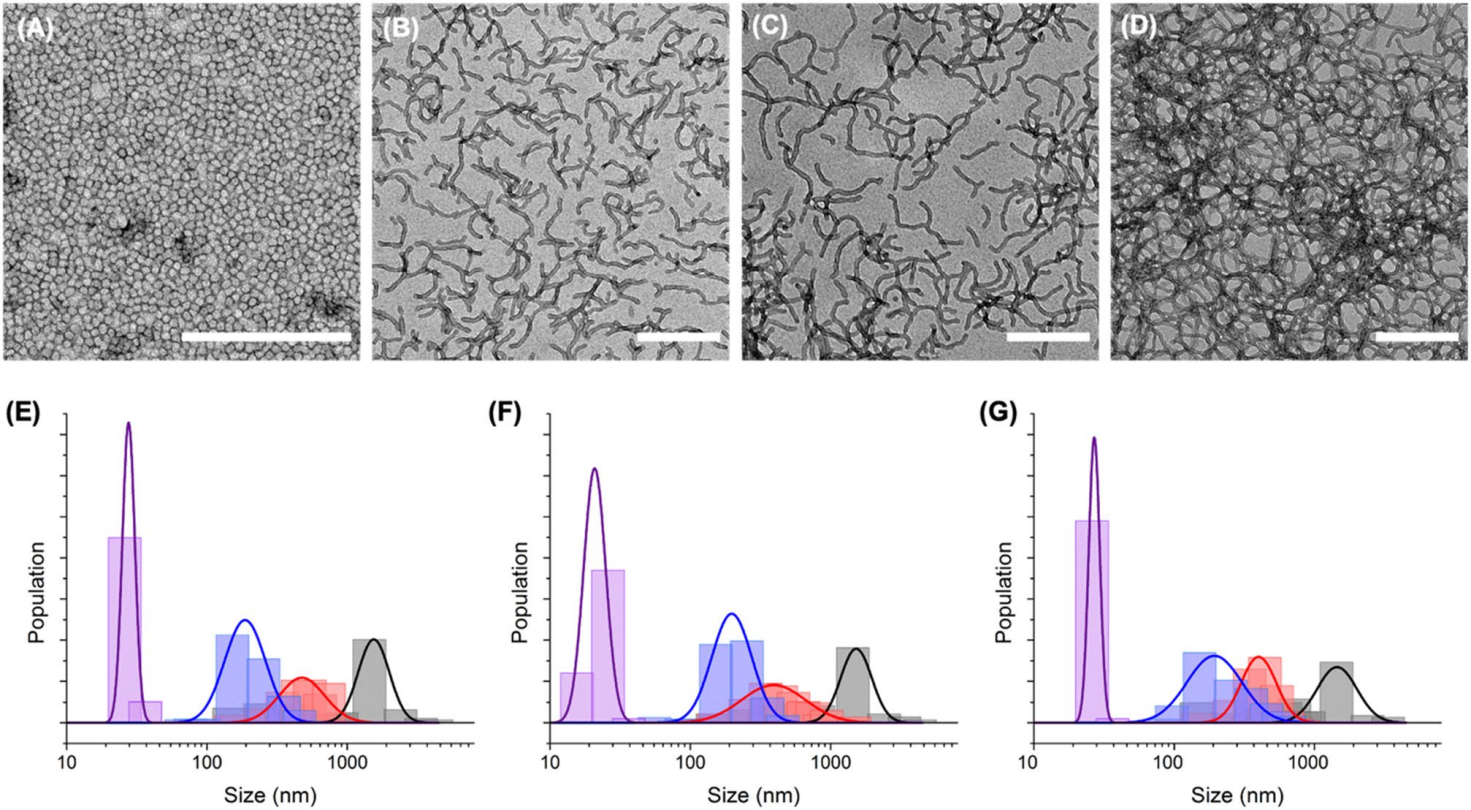
TEM images of colloids (**A**) SM_*NCC*,_ (**B**) SWM_*NCC*,_ (**C**) MWM_*NCC*,_ (**D**) LWM_*NCC*_. Scale bars are 500 nm. Size distribution of the colloids prepared with increasing crosslinking density, (**E**) uncrosslinked colloids (NCC), (**F**) low core crosslinked colloids (LCC), and (**G**) high core crosslinked colloids (HCC) for (purple) spherical colloids (SM), (blue) short worm-like colloids (SWM), (red) medium worm-like colloids (MWM), and (black) long worm-like colloids. The bars in the histograms represent the measurement of the colloid population obtained (See Supporting Information), and the solid lines represent fits of the population measured to a log-normal distribution.

Polymer micelles were used as the template for forming the polymer nanoparticles, which were subsequently used as building blocks for the preparation of colloidal gels. Spherical and worm-like micelles were prepared using reversible addition-fragmentation chain transfer (RAFT) polymerization via polymerization-induced self-assembly (PISA) (Fig. 1). First, a macro-RAFT agent, poly(2-(dimethylamino)ethyl methacrylate) (PDMA), with an average degree of polymerization of 31 units, was prepared (Figure S1). The macro-CTA was then chain-extended by the reaction with a monomer mixture composed of benzyl methacrylate (BzMA) and 2-(methacryloyloxy)ethyl acetoacetate (AAEM) in ethanol. The resulting block copolymer PDMA-*b*-P(BzMA-*co*-AAEM) underwent aggregation in ethanol, leading to the formation of micelles with a P(BzMA-*co*-AAEM) core stabilized by the PDMA segments (Fig. 2). Tuning the polymerization conditions during the synthesis of the second block led to the control over the micellar structure; either spherical micelles (SM), worm-like micelles (LWM) or vesicles were obtained as observed by transmission electron microscopy (TEM) analysis (Figures S2 and S3) when the degree of polymerization of the second block increased.

The worm-like micelles synthesized using PISA (LWM, PDMA_31_-PBzMA_75_) were, on average, ca. 1700 ± 400 nm in length by ca. 25 ± 2 nm in diameter. To further control the length of the worm-like micelle, the long worm-like micelles obtained after the synthesis were subjected to ultrasound. The high local shear stress induced in the suspension during sonication can be used to efficiently cut the micelles, as demonstrated with other block copolymer worm-like micelles^33^. The ultrasonication of the worm-like micelles decreased the average length of the micelles, while the diameter remained constant. During ultrasonication, the implosion of cavitation bubbles generates pressures as high as 2000 atm resulting in localized (both in time and in space) high shear strain in the suspension which can break the micelles. Still, the resulting shorter particles are stable once the sonication is halted. Consequently, the average aspect ratio of the micelles decreased with increasing sonication time from 69 for the as-synthesized micelles to 24 and 9 after 90 and 300 s of sonication, respectively (Table 1, Fig. 2, Figure S4). The particles were identified as long worm-like micelles (LWM) when unsonicated, medium worm-like micelles (MWM) for the micelles sonicated for 90 s, and short worm-like micelles (SWM) for the samples sonicated for 300 s.

**Table 1:** Characterization of the nanoparticles prepared.

NP_X_	Composition	Length (nm)^a^	PDI^b^	Aspect ratio
SM_*NCC*_	PDMA_31_-PBzMA_40_	25	0.03	1
SWM_*NCC*_	PDMA_31_-PBzMA_75_	220	0.12	9
MWM_*NCC*_		590	0.25	24
LWM_*NCC*_		1700	0.06	69
SM_*LCC*_	PDMA_31_-PBzMA_35_-co-PAAEM_5_	22	0.03	1
SWM_*LCC*_	PDMA_31_-PBzMA_70_-co-PAAEM_5_	230	0.11	9
MWM_*LCC*_		590	0.31	24
LWM_*LCC*_		1670	0.07	68
SM_*HCC*_	PDMA_31_-PBzMA_30_-co-PAAEM_10_	26	0.22	1
SWM_*HCC*_	PDMA_31_-PBzMA_65_-co-PAAEM_10_	260	0.22	11
MWM_*HCC*_		550	0.29	19
LWM_*HCC*_		1700	0.11	70

X represents the crosslinking density of the micelle’s core. NCC = No core crosslinking (crosslinkable monomer = 0 mol%), LCC = low core crosslinking (crosslinkable monomers = 6.7 mol%), HCC = high core crosslinking (crosslinkable monomer = 13 mol%).

^a^Calculated from the log-normal distribution of the size measured by TEM for N > 75.

^b^Calculated from the log-normal distribution of the size measured by TEM for N > 75 as PDI = (σ/μ)^2^ with σ the standard deviation of the distribution and μ the mean of the distribution.

During the synthesis of the second block of the copolymer, a mixture of BzMA and AAEM was used. PBzMA is poorly soluble in ethanol, the solvent used during the synthesis, and was the main responsible for the formation of the micelles. The second monomer, AAEM, was added to the hydrophobic block to allow the post-polymerization crosslinking of the resulting micelles. AAEM can react with a diamine to produce a crosslinked network and stabilize the micelles via the covalent crosslinking of the core.

In order to tune the rigidity of the micelle core, the ratio of AAEM in the feed of BzMa and AAEM used during the synthesis of the micelle core was increased from 0 to 6.7 and 13 mol%. The acetoacetate groups of the AAEM can rapidly react with an amine under mild conditions to form imines^34^. Therefore, using a diamine such as diaminopropane, it was possible to crosslink the hydrophobic segments in the core of the micelles. The reaction in the core of the micelles using diaminopropane yielded the corresponding nanoparticles in ethanol. The reaction between AAEM and diaminopropane was almost quantitative, as followed by NMR spectroscopy (Figure S5 and Table S1), and the resulting nanostructures were stabilized by the formation of a covalent crosslinked imine network and could be easily redispersed in a solvent like THF that would otherwise dissolve the uncrosslinked micelles (Figure S6). The nanoparticles were transferred to water after core crosslinking, and their morphologies were measured using TEM (Fig. 2). The samples were identified either as NCC (no core crosslinking) for the micelles prepared without AAEM, LCC (low core crosslinking) for the micelles prepared using ca. 6.7 mol% of AAEM and HCC (high core crosslinking) for the particles prepared with 13 mol% of AAEM.

**Figure 3:**
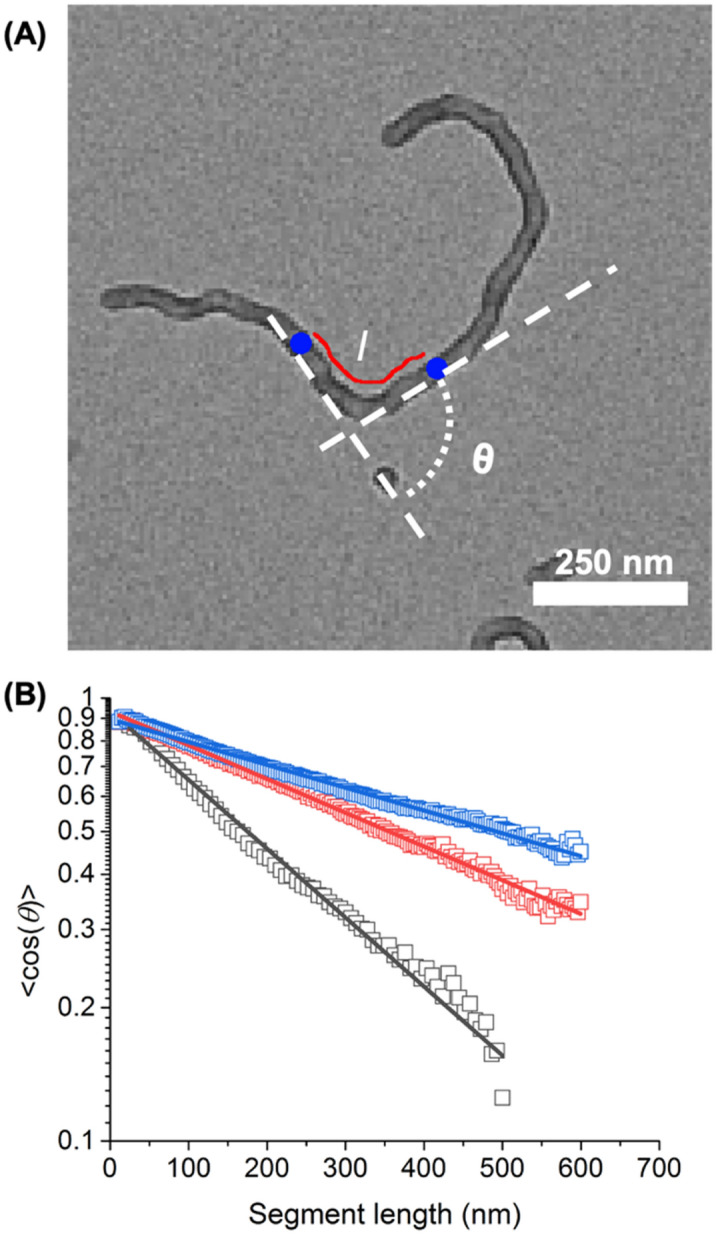
Persistence length of the micelles and crosslinked particles. (**A**) Parameter for the determination of the persistence length. (**B**) Decay of the tangent-tangent correlation for LWM_*NCC*_ (Black), LWM_*LCC*_ (Red), LWM_*HCC*_ (Blue). The lines represent fits to Eq. 1. The data in B represent the average of the decay curves for 326 NCC, 133 LCC, and 258 HCC worm micelles, respectively.

The crosslinking reaction did not affect the size and distribution of the resulting colloids (Fig. 2 and Figure S7). The core crosslinking of the micelles stabilized the colloids, but also tuned the stiffness of the particles. To evaluate the stiffness of the colloids, the persistence length (*P*) of MWM and LWM was calculated using the decay of the orientation correlation within the worm micelle^35–37^:

1 $$<\mathrm{cos}\left(\theta \right)>={e}^{\frac{-l}{sP}}$$

where θ is the angle between the tangents of two points of the micelle separated by a contour segment of length *l* as described in Fig. 3 and *s* a structure parameter set to 2 for object equilibrated on a 2D substrate. The persistence length obtained with Eq. 1 can be used to evaluate the stiffness of the colloid with:

2 $$P=\frac{B}{{k}_{b}T}$$

where *B* is the flexural rigidity of the colloids, *k*_b_ is the Boltzmann constant, and *T* the temperature.

The persistence length increased from 128 ± 3 nm for LWM uncrosslinked micelles to 253 ± 4 nm LWM_*LCC*_ crosslinked particles and 331 ± 6 nm for the LWM_*HCC*_ particles. No differences were observed between the MWM and the LWM, having the same chemical composition. Given that the persistence length is directly proportional to the bending stiffness, the result suggested that the moderately crosslinked colloids were twice as stiff as the uncrosslinked colloids and the heavily crosslinked colloids were 2.8 times more rigid than the uncrosslinked colloids. The rigidity of the colloids was respectively 0.5 gN·nm^2^ for uncrosslinked micelles (NCC), 1.0 gN·nm^2^ for moderately crosslinked particles (LCC) and 1.4 gN·nm^2^ for heavily crosslinked particles (HCC).

**Figure 4:**
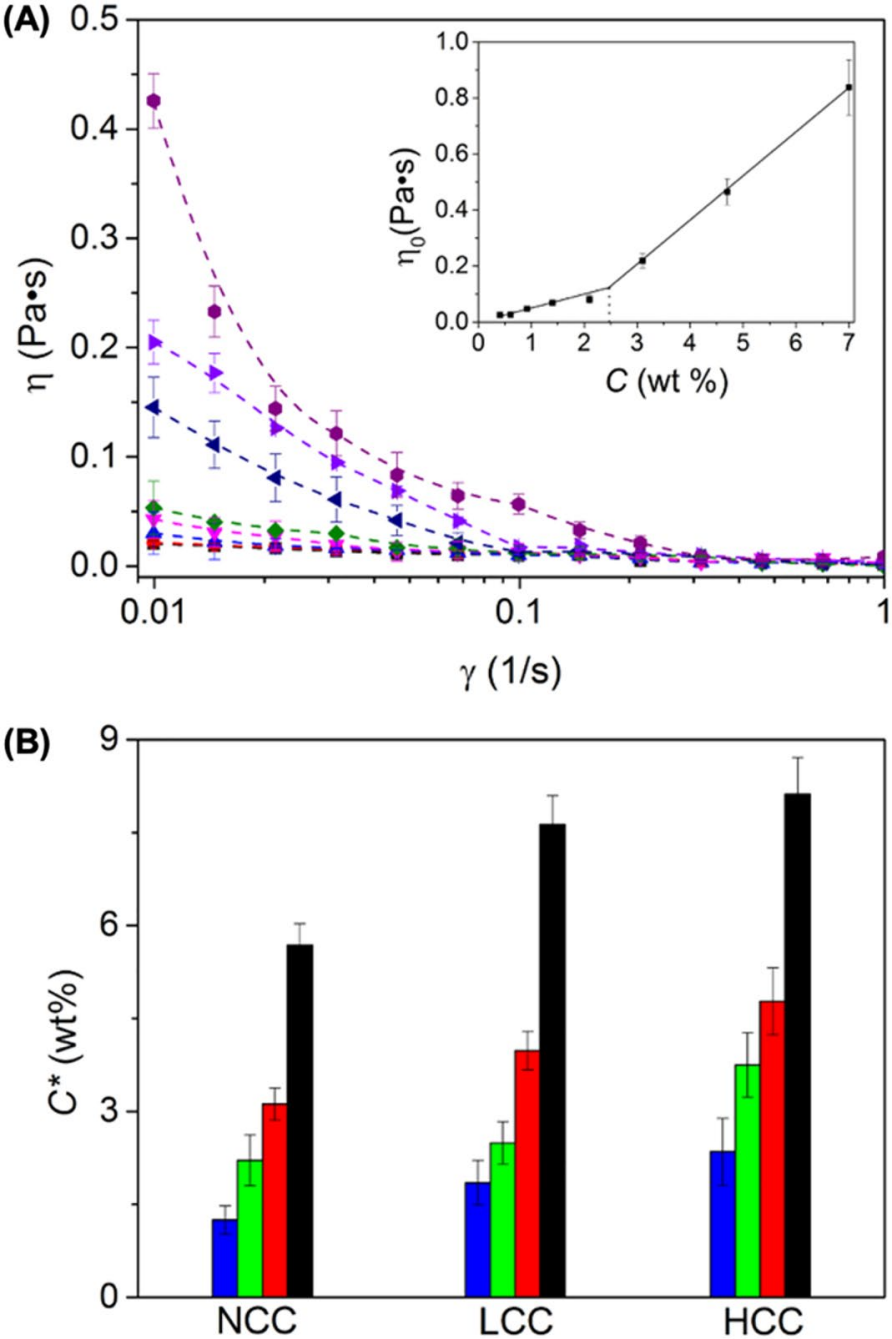
(**A**) Dynamic viscosity of suspensions of LWM_*NCC*_ at concentrations ranging from 0.4 to 7.0 wt%. The inset represents the increase in the zero-shear viscosity (η_0_) of the suspensions with the concentration of the colloids in suspension. (**B**) Influence of architecture and rigidity on the transition between the dilute and semi-dilute regime. Sphere (black); Short worm (red); Medium worm (green); Long worm (blue) for the uncrosslinked colloids (NCC), colloids with low crosslinking density (LCC) and colloids with high crosslinking density (HCC).

The rheological properties of the LWM_*NCC*_ colloidal suspensions were studied with increasing solid content in water under steady shear conditions (Fig. 4A, Figure S8). The viscosity displayed two distinct regimes of shear-dependent behavior. At low concentration, the viscosity of the suspensions increased moderately with the colloid concentration and displayed a Newtonian behavior. At higher concentration, the viscosity of the suspensions increased more steeply with increasing concentration, and all the suspensions displayed a shear-thinning behavior. This transition between the dilute and semi-dilute regime occurred at a critical concentration C* where the colloids started to interact with each other, whether through the interaction of the PDMA layer of adjacent colloids or through colloid-colloid interaction. The critical concentration C* was defined as the inflection point in the plot of the variation of the zero-shear viscosity with the particle concentration.

Interestingly, the value of C* was influenced by the aspect ratio and by the stiffness of the colloidal particles studied. For a given concentration, the viscosity of the suspension systematically decreased as the aspect ratio of the colloids decreased. In addition, the critical concentration C* required to observe the transition between the dilute and semi-dilute regime increased when the aspect ratio of the colloid decreased. This behavior can be attributed to the fact that anisotropic nanoparticles have a larger surface area volume in comparison to a sphere, which favors attractive van der Waals interaction between the colloids. Moreover, increasing the aspect ratio of the nanoparticle created a larger excluded volume around the colloids resulting in a higher viscosity^38^. Furthermore, colloids with higher aspect ratios displayed a more pronounced shear-thinning behavior due to the alignment of the colloid in the direction of the force applied as the sample was subjected to shear. In addition, at a given concentration, when the stiffness of the colloid increased, the viscosity moderately decreased. Furthermore, as the stiffness of the colloid increased, the value of C* increased for colloids of a specific length. Those results indicated that as the colloid became stiffer, the colloid–colloid interactions decreased (Fig. 4B), and the worm-like colloids might not entangled as efficiently with the increased rigidity.

**Figure 5:**
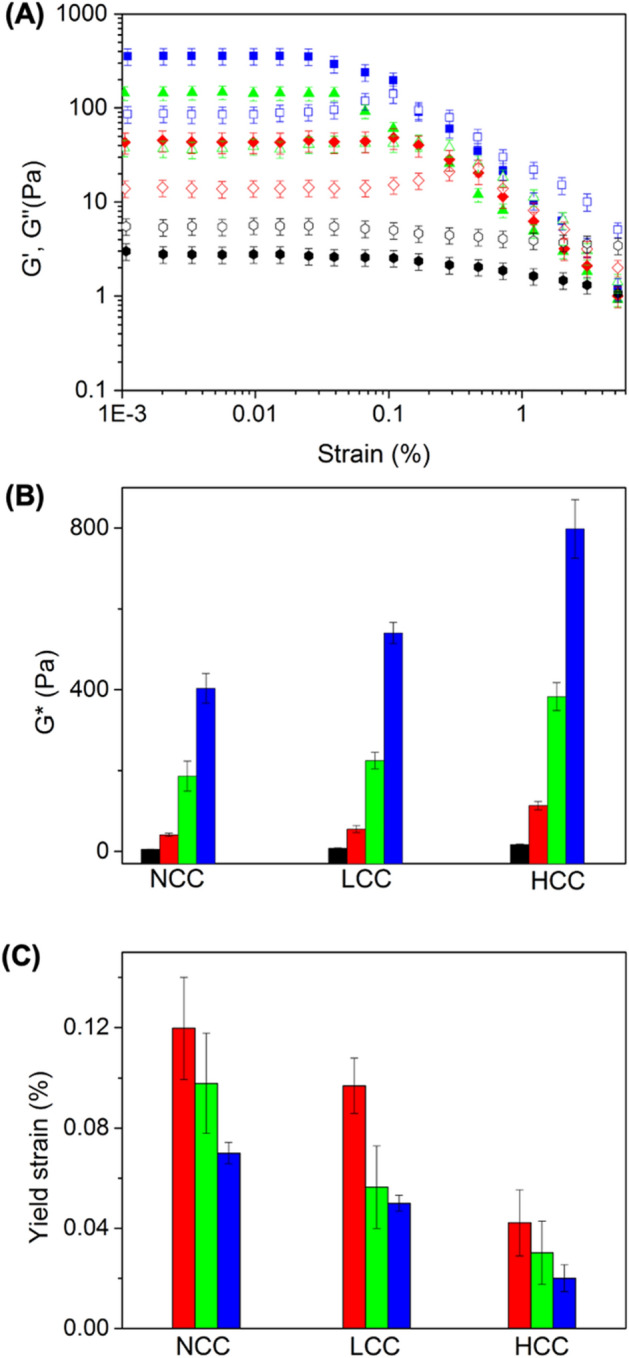
Viscoelastic behavior G' (filled symbols) and G'' (open symbol) of 10 wt% suspensions of uncrosslinked colloids in water. (**A**) Effect of the aspect ratio of micelles, (**B**) Effect of rigidity and aspect ratio on the complex dynamic modulus (G*) of the colloids, and (**C**) yield strain of the colloidal gels. Sphere (Black); short worm (Red); medium worm (Green) and long worm (Blue).

At high concentrations (well above C*), the presence of increased colloid–colloid interaction resulted in a transition from a liquid-like to gel-like behavior. The gel-like behavior of concentrated suspensions (10wt%) was analyzed by dynamic rheology (strain-sweep (Figure S9) and frequency sweep (Figure S10)). At a concentration of 10wt% of colloids in suspension, the worm-like particles SWM, MWM, and LWM with different degrees of crosslinking and rigidity formed gels, and the elastic modulus (G’) was higher than the viscous modulus (G”). However, the suspension of spherical colloids displayed a liquid-like behavior (Fig. 5A), where G” was systematically larger than G’.

The dynamic rheology of the colloidal gels formed the worm-like particle with different aspect ratios displayed a linear-viscoelastic region with a higher elastic modulus than the viscous modulus at low deformation in every case. The average complex modulus (G*) of the different gels increased with increasing aspect ratio of the colloidal building block. Given that the total volume occupied by the colloids in the gel was similar for all the suspensions prepared with colloids with different aspect ratios, this increase in the colloid-colloid interactions cannot be directly ascribed to the interdigitation of the water-swelled PDMA canopy. Rather, the arrested gel-like behavior must be, at least partially, ascribed to the geometry of the micellar core. An increase in the aspect ratio of the colloids led to the formation of a more cohesive network, either because of the increased excluded volume of higher aspect ratio colloids or through enhanced colloid-colloid interaction due to a combination of higher surface area and the potential occurrence of worm-worm entanglements.

Furthermore, when the colloidal gels were prepared with colloids having the same aspect ratio but different stiffness, an increase in the complex modulus (G*) of the resulting gels was observed as the stiffness of the building block increased. Previous works suggest that the stiffening of the worms particles through core crosslinking can increase the modulus of the gels formed^39,40^. Here, when comparing LWM, the G* increased from 380 Pa for LWM_NCC_ to 581 Pa for LWM_LCC_, and 813 Pa for LWM_HCC_ (Fig. 5B). These results represented the direct comparison between the stiffness of colloidal gels and the rigidity of the colloidal particles used as building blocks. As the crosslinking of the colloids increased, the persistence length was shown to decrease (Fig. 3), which would reduce the opportunity for the formation of entanglement between colloids. When the rigidity of rod-like objects in suspension increases, the relaxation time of the network formed decreases^41^. Hence, each crosslinking point of the network formed by the colloidal interaction through entanglement and interparticle interaction remained dynamic in nature, but existed for longer periods of time, leading to the formation of a less transient network, and, consequently, the mechanical properties of the network increased.

In addition, the fragility of the colloidal gels, defined by the yield strain ϒ_c_, was measured (Fig. 5C). The results show that the yield strain of the gels was affected by the aspect ratio and rigidity of the colloids. The suspensions of spheres did not form gels in the concentration range studied. The gels formed with WM were stiffer (higher G*) and more fragile (lower yield strain) with the increase of aspect ratio. Moreover, comparing gels prepared with colloids having different rigidity showed that the yield strain decreased when the rigidity of the colloids increased. The gels prepared with the higher crosslinking density were the stiffest and the most fragile. When the rigidity of the core of colloids increased, the suspensions behaved in a more solid-like manner, and these suspensions were tougher but displayed lower yield strain.

The result obtained during the rheological analysis of the suspensions of colloids with different aspect ratios and rigidity revealed the effects of the nanocolloids characteristics on the properties of the resulting colloidal gels. For colloids with a given rigidity, increasing their aspect ratio resulted in the formation of a colloidal gel at a lower concentration, C*, (Fig. 4), for the HCC particles, an increase in the aspect ratio of the nanocolloid from 1 to 70 resulted in a decrease of ca 70% in the value of C*. This behavior could be ascribed to either the larger surface area of the particle or the increased probability for long worm-like particles to entangle. The critical concentration at which the sol–gel transition was observed was also influenced by the rigidity of the colloids; for a given aspect ratio, C* increased with increasing the rigidity; when considering the LWM nanocolloids, an increase in the rigidity of the particles from 0.5 to 1.4 gN nm^2^ resulted in an increased in C* of ca. 90%. More rigid particles appeared to interact less efficiently with each other, either due to a decrease in the entanglements between the worm or a reduction in the interpenetration and entanglements between the canopy of end-tethered polymer chains on adjacent nanocolloids. This behavior would be in keeping with the reduced segmental mobility observed for polymer chains grafted on rigid polymer nanoparticles^42^.When comparing suspensions containing the same concentration of polymer nanoparticles, the complex modus of the gel increased with increasing aspect ratio and rigidity. For highly crosslinked colloids, increasing the aspect ratio from 1 to 70 resulted in an increase in G* of ca. 4500%; similarly, increasing the rigidity of the colloids from 0.5 to 1.4 gN nm^2^ resulted in a rise in G* of ca. 98%. As the gels became stiffer, they also became more fragile and displayed lower yield strain with increasing aspect ratio and rigidity. For highly crosslinked colloids, increasing the aspect ratio from 1 to 70 resulted in a decrease in ϒ_c_ of ca. 53%; similarly, increasing the rigidity of the colloids from 0.5 to 1.4 gN nm^2^ resulted in a drop in ϒ_c_ of ca. 72%.

**Figure 6:**
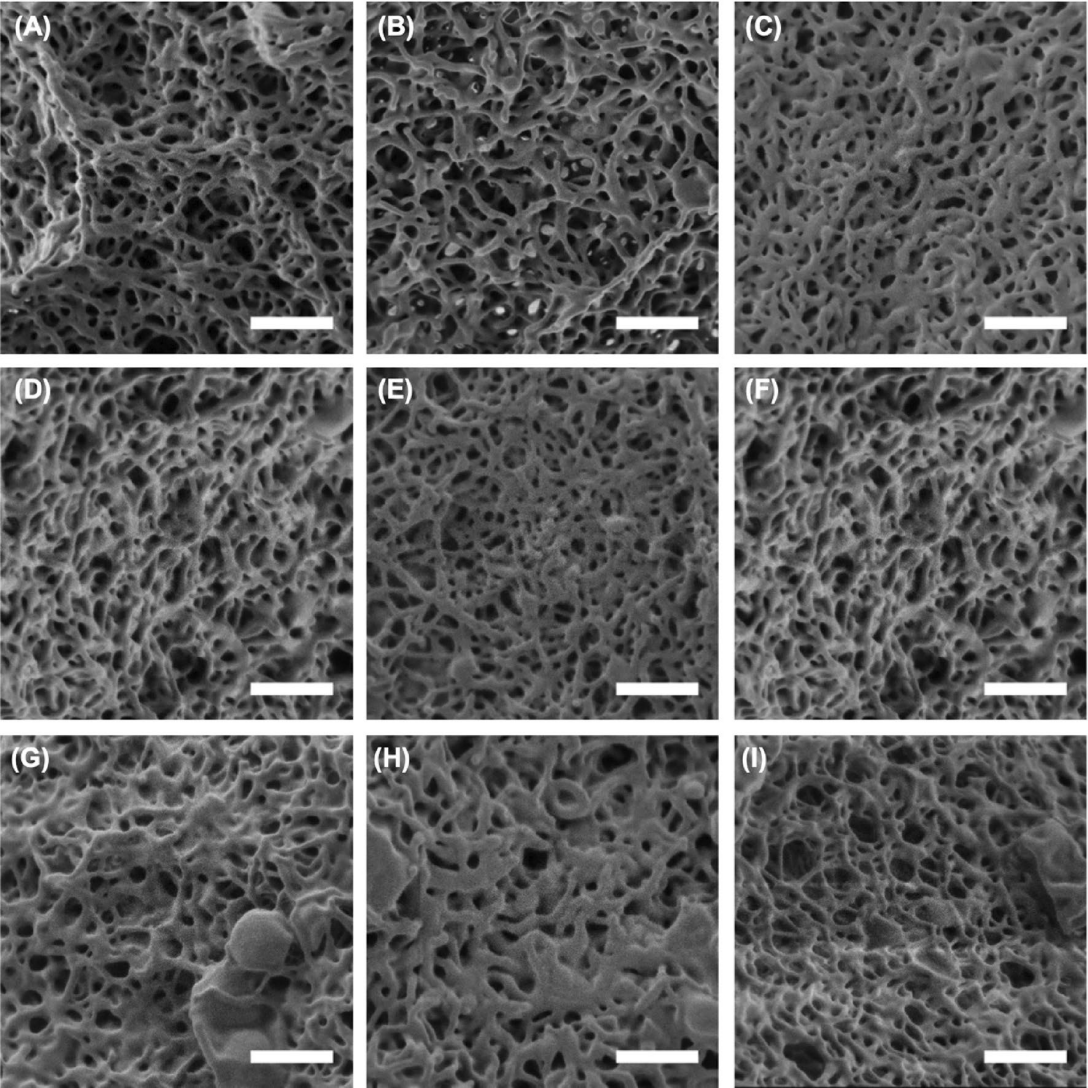
SEM images of the microstructure of the colloidal gels prepared with different colloidal building blocks. Gels prepared at room temperature by 10 wt% suspensions of uncrosslinked colloids with different aspect ratios: SWM_*NCC*_ (**A**), MWM_*NCC*_ (**B**) and LWM_*NCC*_ (**C**), Gels prepared at room temperature by 10 wt% suspensions of highly crosslinked colloids with different aspect ratios: SWM_*HCC*_ (**D**), MWM_*HCC*_ (**E**) and LWM_*HCC*_ (**F**) Gels prepared at 45 °C by 1.0 wt% suspensions of uncrosslinked colloids with different aspect ratios: and SWM_*NCC*_ (**D**), MWM_*NCC*_ (**E**) and LWM_*NCC*_ (**F**). The scale bars are 500 nm.

To ensure that the modification of the mechanical behavior observed by rheology originated from the mechanical properties of the colloids and not from the microstructure of the gels obtained during the association of the nanocolloids, scanning electron microscopy (SEM) was performed. Figure 6 shows representative SEM images of the nanofibrillar structure of the colloidal gels. The mean fiber diameter and the apparent pore size observed were not affected (in a statistically significant manner) by the length nor the rigidity of the colloids (Figure S11). Thus, the variation in the mechanical properties of the gels can only be ascribed to the properties (aspect ratio and rigidity) of the colloidal building block, not to the type of structure formed in the colloidal suspension.

The state of the swollen PDMA canopy also had a critical influence on the rheological properties of the colloidal gels. Free PDMA chains in aqueous solution displayed a typical coil-to-globule transition above their lower critical solution temperature (LCST) around 35 °C. Upon heating, the water-soluble hydrated chains of the PDMA will collapse to form compact deswollen globules^43,44^. When the PDMA chains were immobilized at the surface of the nanoparticles, upon heating, the colloidal system displayed a lower critical aggregation temperature (LCAT) in place of an LCST. When a suspension of colloids at a concentration of 0.01 wt% was heated above LCAT, the aggregation of the colloids was observed (Figure S12). DLS analysis of the 0.01 wt% suspension of spherical particles at 25 and 45 °C show the formation of aggregates; at 25 °C the spherical particle displayed a size of 23 nm, which increased to ca. 800 nm at 45 °C. This change in size was fully reversible and can be attributed to the deswelling of the PDMA chains above LCAT, which reduced the sterical repulsion between adjacent colloids and induced the formation of small aggregates. When heating the colloids above the LCAT, the structure of the colloids in suspension remained the same (Figure S13) only their aggregation was observed.

**Figure 7:**
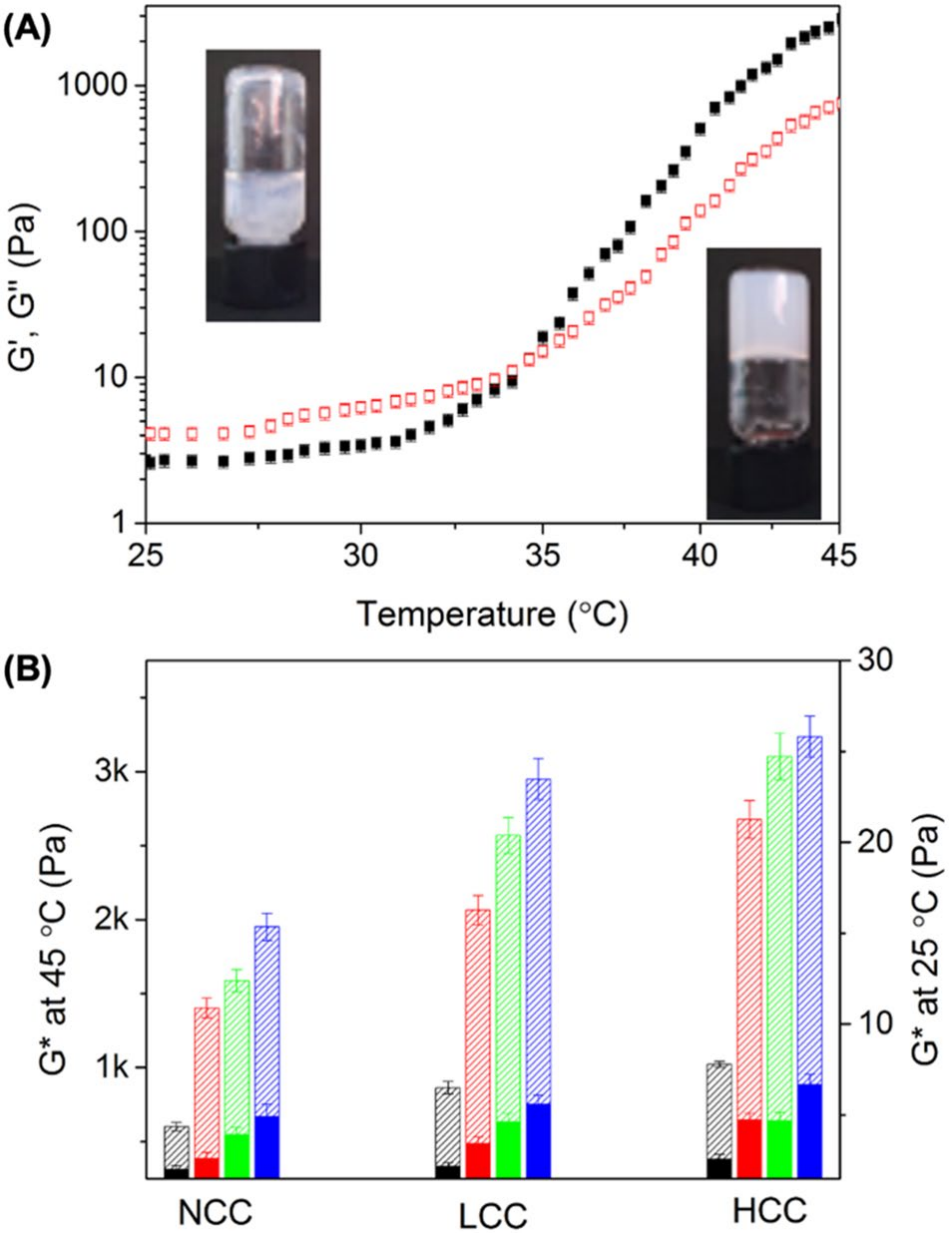
(**A**) Temperature dependence of storage modulus G' (solid symbols) and loss modulus G'' (open symbols) of a 1.0 wt% suspension of uncrosslinked spherical colloids. (**B**) Effect of the aspect ratio and rigidity of the colloids on the properties of the thermally-induced colloidal gels on the modulus of the suspensions at 25 °C (solid bars) and 45 °C (hashed bars), Sphere (Black); Short worm (red); Medium worm (green); Long worm (blue).

When the concentration of colloids in suspension was increased to 1 wt%, increasing the temperature of the suspensions above LCAT led to the formation of colloidal gels (Fig. 7). The formation of the gels was fully reversible (Figure S12). Figure 7A shows that the diluted suspension of colloidal nanoparticles behaved like a liquid at room temperature (G" > G'), and as the temperature of the suspension increased, the gelation of the suspension occurred (G' > G"). The desolvation of the PDMA chains led to the formation of an extended network of interacting colloids. During the formation of this colloid network, no gelation shrinkage was observed, and all the water present in the suspension below LCAT was efficiently entrapped in the colloidal gel. The same behavior was observed for all the colloidal suspensions (Figure S14).

The colloidal gels obtained by thermally-induced gelation (Fig. 7) were more rigid than those prepared at room temperature and with higher solid content (Figs. 4 and 5). Furthermore, the mechanical properties of the resulting colloidal gels were affected by the rigidity of the colloid and their aspect ratio. More rigid colloidal building blocks resulted in the formation of stiffer colloidal gels. Similarly, the colloids with the highest aspect ratio also formed stiffer colloidal gels (Fig. 7B). The differences in the mechanical properties of the thermally-induced gels and the concentration-induced gels can be ascribed to the mechanical properties of the colloids. The deswelling of the PDMA chain not only increased the hydrophobicity of the colloids, but also increased the rigidity of the colloids since a deswollen polymer network usually has a higher young modulus than a swollen polymer network^45^. Overall, during thermally-induced gel formation, the nanocolloidal network became more persistent because of the stronger contacts between the colloidal nanoparticles, hence providing an enhanced opportunity for inter-particle interaction.

## Conclusion

The study of colloidal suspensions containing building blocks with the same composition and occupying similar volume fraction but with distinct rigidity and aspect ratio reveal new insights into the formation of colloidal gels. Suspensions of all particles displayed a non-Newtonian behavior. However, the viscoelastic behavior of all suspensions depended on the concentration of the colloids and the rigidity and aspect ratio of the colloidal building blocks. The critical concentration at which the colloidal suspensions started to behave as a colloidal gel shifted to a higher concentration with increasing rigidity and aspect ratio. As the concentration increased, the colloidal particles formed fibrillar gels, likely because of colloid-colloid interaction and the potential entanglement between particles. The formation of fibrillar gel occurred more easily with nanocolloids having a large aspect ratio because the larger surface area of the colloids resulted in more efficient particle–particle interactions, and large and flexible colloids can entangle more efficiently. On the one hand, the key effect on the critical concentration where the sol–gel transition was observed was the aspect ratio of the colloids; on the other hand, the stiffness of the resulting gels was mainly influenced by the rigidity of the colloids. The mechanical properties of the gels were affected by both the aspect ratio and rigidity of the colloids. The longer and more rigid colloids displayed the highest modulus, but also the lowest yield strain. Finally, the suspensions of these colloids also exhibited a thermally-induced sol–gel transition at low concentrations. The thermally-induced gelation led to the formation of tougher gels than the gels formed at room temperature. Nevertheless, the influences of the aspect ratio and the crosslinking of the core of the colloids on the properties of the thermally-induced gel was similar to what was observed with the gels prepared at room temperature by concentration-induced gelation. The fibrillar gels formed here could potentially find applications in strengthening and guiding the regeneration of load-bearing soft tissues because they display an interesting range of moduli relevant to the culture of soft tissues.

## Method

### Colloid synthesis

First, 2-(dimethylamino)ethyl methacrylate (DMAEMA) 2-(methacryloyloxy)ethyl acetoacetate (10.0 g) was polymerized via reversible addition-fragmentation chain-transfer (RAFT) polymerization using 4-cyano-4-(2-phenylethanesulfanylthiocarbonyl) sulfanylpentanoic acid (0.432 g) as the chain transfer agent (CTA), 4,4′-azobis(4-cyanovaleric acid) (36 mg) as the initiator and THF (10 g) as a solvent, see SI for details. After 6.5 h of polymerization at 65 °C, the polymerization was stop and the polymer recovered and purified. The resulting polymer (PDMA_31_-CTA) was composed on average of 31 units of DMAEMA and was used as the macro-CTA for the polymerization of a mixture of benzyl methacrylate (BzMA) and 2-(methacryloyloxy)ethyl acetoacetate(AAEM). The conditions of the RAFT polymerization was tuned to produce polymer with different length (see SI for details). In a typical reaction, 144 mg of PDMA-CTA is combined with 0.184 g of BzMA and 0.032 g of AAEM dissolved in 1.05 g of ethanol at 70 °C. The addition of 2,2’-azobis(isobutyronitrile) (0.9 mg) initiated the reaction. After 24 h, the resulting polymer colloids were collected and purified by dialysis. While BzMA is miscible with ethanol, poly(BzMA) is not. The resulting block copolymers precipitated during the synthesis forming polymer aggregates by this polymerization-induced self-assembly (PISA) process. of the PDMA-b-P(BzMA-co-AAEM) was observed. Tuning the length of the P(BzMA-co-AAEM) block (see SI for details) allowed to control the shape of the polymer colloids formed. The synthesis of PDMA_31_-b-P(BzMA_35_-co-AAEM_5_) described resulted in the formation of worm-like micelles.

### Control of the length of the micelles

A total of 1.0 g of the worm micelles was diluted to 3.25 wt% by the addition of ethanol. The suspension was sonicated for 90 s or 5 min in pulses of 3 seconds on and 2 seconds off using a Branson 450D sonoprobe equipped with a 3 mm tapered micro-tip

### Covalent crosslinking of the core of the polymer micelles

To 50 mL of a 3.25 wt% suspension of polymer micelles in suspension in ethanol, 25 μL of 1,3-diaminopropane was added. The reaction between AAEM and diamino propane occurred at room temperature. After 24 h the resulting crosslinked micelles were purified by dialysis against ethanol.

### Rheology

Rheological measurements were performed on a Bohlin Gemini rheometer with aqueous suspension of the polymer colloids. Continuous shear experiments were performed by increasing the shear rate from 0.01 to 1 Hz. Dynamic measurements were performed either at a fixed frequency of 1 rad/s while increasing the deformation from 0.01 to 500% or at a fixed stain of 1% with a frequency varying between 0.001 and 100 rad/s.

## Supplementary Information

Supplementary Information.

## Data Availability

The data are available from the corresponding author upon request.
