# Supplementary material for: Behavior of colloidal gels made of thermoresponsive anisotropic nanoparticles
Source: Sci Rep. 2022 Jul 15;12:12157. doi: 10.1038/s41598-022-16414-w (PMC9287383; doi:10.1038/s41598-022-16414-w)
Supplement: Supplementary file 1 — Supplementary Information. [file 41598_2022_16414_MOESM1_ESM.pdf]

Behavior of Colloidal Gels Made of Thermoresponsive Anisotropic Nanoparticles

Long Yang, [†,‡] Héloïse Thérien-Aubin[‡,§,*]

[†] School of Textile Science and Engineering, Xi'an Polytechnic University, China

[‡] Max-Planck Institute for Polymer Research, Mainz, Germany

[§] Department of Chemistry, Memorial University of Newfoundland, Canada

*Correspondence: htherienaubin@mun.ca

EXPERIMENTAL SECTION

Materials

The monomers 2-(dimethylamino)ethylmethacrylate (99%, DMAEMA), benzyl methacrylate (98%, BzMA) and 2-(methacryloyloxy)ethyl acetoacetate (95%, AAEM) were purchased from Sigma-Aldrich and purified on an alumina oxide column. 4-Cyano-4-(2-phenylethanesulfanylthiocarbonyl) sulfanylpentanoic acid was synthesized as previously reported.[1] 4, 4'-Azobis(4-cyanovaleric acid) (ACVA)) or 2,2'-azobis(isobutyronitrile) (AIBN) were recrystallized twice before use.

^{1}H NMR Spectroscopy

All NMR spectra were recorded on a Bruker Avance 300 MHz.

Dynamic Light Scattering (DLS)

Dynamic light scattering measurements were performed using a Malvern Zetasizer Nano S90 using a He‒Ne laser (633 nm) and analysis angle of 90°. The hydrodynamic diameter of the polymer structures was measured in water. All samples were filtered with 0.45 μm filter before measurements.

Transmission Electron Microscopy (TEM)

The nanostructures were analyzed using a JEOL-1400 transmission electron microscope at an accelerating voltage of 120 kV. Typically, TEM grid preparation was as follows: the samples were diluted with Milli-Q water to approximately 0.05 wt %. The TEM grid was dipped in the diluted solution, the excess solution was blotted with a filter paper, and the grid was then dried at room temperature. To stain the deposited nanoparticles, a 0.4% w/w aqueous solution of uranyl acetate was placed via micropipette on the grid for 20 s and then carefully blotted to remove excess stain.

Image analysis

The TEM images were analyzed using MATLAB. First, the TEM micrographs were converted into binary images. The build-in function regionprops was used to identify the different objects present in the image. Then, the image was skeletonized (bwmorph) to identify branched or overlapping worms. The end-to-end distance was measured along each skeleton (bwdistgeodesic). The cosinus of the angle between two tangents of the worms was calculated as the dot product of the tangent vectors divided by the product of their lengths. The values reported are the average of all the particles measured (N>75).

Rheological Measurements

Rheological measurements were carried out on Bohlin Gemini rheometer equipped either with coaxial cylinder geometry with a measuring bob radius of 25 mm, a measuring cup radius of 27.5 mm and 13.0 mL sample volume or a cone and plate geometry (1° cone angle and 40 mm diameter with a truncation gap of 500 μm). The viscosity of the suspension was measured in shear-sweep experiment from 0.01 to 1 Hz and used to extrapolate the zero-shear viscosity of each sample. For the dynamical measurements, about 5 mL of gel was loaded onto the plate, and the cone was lowered to minimize the truncation gap, and the excess of gel was removed. Before experiments, samples were equilibrated in the geometry for 10 min. Oscillatory strain sweep measurements were run from 0.01 to 500% deformation at a fixed frequency of 1 rad/s. Oscillatory temperature sweep measurements were performed at different temperatures at a frequency of 1 rad/s, with a constant strain of 1%, which is within the linear viscoelastic regime of the hydrogels. Temperature for the frequency step was maintained from 25 to 45 °C for each frequency sweep.

Synthesis of Poly(2-(dimethylamino)ethyl methacrylate) $PDMA_{31}$-CTAs

The chain transfer agent 4-cyano-4-(2-phenylethanesulfanylthiocarbonyl) sulfanylpentanoic acid (0.432 g, 1.27 mmol), the monomer DMAEMA (10.0 g, 63 mmol) were added to THF (10 g) with ACVA (36 mg, 0.127 mmol), the solution was degassed with argon at ice bath for 0.5 h and then immersed into an oil bath at 65 °C. After 6.5 h the polymerization was quenched using an ice bath. The monomer conversion was analyzed by ^{1}H NMR spectroscopy. PDMA was purified by precipitation into cold hexane three times and dried under vacuum to yield a yellow product. The PDMA macro-CTAs were characterized by ^{1}H NMR spectroscopy and GPC.

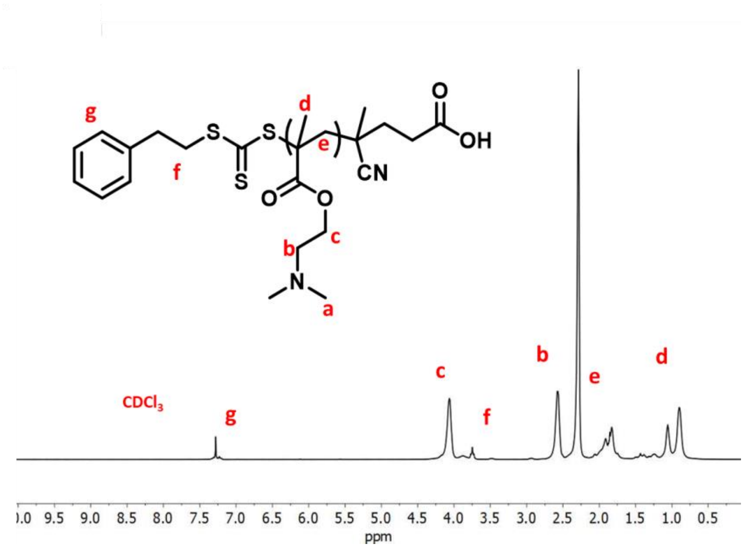

Figure S1. ^{1}H NMR spectra of Poly(2-(dimethylamino)ethyl methacrylate) in $CDCl_3$.

Synthesis of micelles ($PDMA_{31}$-b-$PBzMA_x$) via Dispersion Polymerization in Ethanol

A typical RAFT dispersion polymerization was conducted at a concentration 15% w/w total solid fraction. First, $PDMA_{31}$-CTA (144 mg, 0.028 mmol), BzMA (1.5 g, 8.4 mmol), and AIBN (0.90 mg, 0.006 mmol) were dissolved in ethanol (9.32 g), the reaction mixture was degassed with argon in an ice/water bath for 20 min, and then placed in a preheated oil bath at 70 °C for 24 h. The length of the PBzMA block was varied by using different amount of BzMA (0.10 g to 1.5 g). According to ^{1}H NMR spectroscopy analysis, the final conversion of BzMA was above 99%. The resulting polymers were analyzed by ^{1}H NMR spectroscopy and GPC.

During the following PDMA-PBzMA diblock copolymer syntheses, the degree of polymerization of the PBzMA block was systematically varied, which allowed to tune the morphology of the micelles.

Synthesis of Crosslinkable micelles ($PDMA_{31}$-b-$PBxMA_{35}$-co-$AAEM_5$)

The synthesis of crosslinkable block copolymer was performed similarly to the synthesis of $PDMA_{31}$-b-$PBzMA_x$ with a mixture $PDMA_{31}$ (0.144 g, 0.028 mmol), BzMA (0.184 g, 1.04 mmol), 2-(methacryloyloxy) ethyl acetoacetate (AAEM) (31 mg, 14 mmol) and AIBN (0.90 mg, 0.006 mmol) in EtOH (1.05 g). The reaction mixture was degassed with argon in an ice/water bath for 30 min, and then placed in a preheated oil bath at 70 °C for 24 h.

In order to control the density of crosslinking, the DP of AAEM of $PDMA_{31}$-b-$PBzMA_x$-co-$AAEM_y$ was varied at 5 and 10, respectively but keeping x+y=40. This was realized by tuning the amount of AAEM and BzMA in the monomer mixture.

Control of the length of the micelles

A total of 1.0 g of the worm micelles was diluted to 3.25 wt% by the addition of ethanol. An ultrasound probe was then placed in the worm solution kept in an ice-bath and the worms were cut to smaller rods at different sonication time. The sonication was applied with pulses of 3 seconds on and 2 seconds off with a Branson 450D sonoprobe working at 20 kHz for a specific period of time (90 s or 5 min) 35% amplitude (ca. 140W) using a 3 mm tapered micro-tip. After ultrasound cutting, the rod-like micelles were characterized by TEM.

Covalent crosslinking of the core of the polymer micelles

1,3-diaminopropane can react with the acetoacetate of the AAEM, located in the core of the micelles, via nucleophilic substitution to form an imine, resulting in the covalent stabilization of the micelles. The crosslinking reactions were conducted at 25 °C using 1,3-diaminopropane to 50 mL of a 3.25 wt% suspension of micelles in ethanol, 25 μL of 1,3-diaminopropane was added and let to react for 24 h. The resulting crosslinked micelles were purified by dialysis against ethanol. The final suspensions were prepared by the redispersion of different concentration of worms and rod shape particle dispersions were prepared after removing ethanol and transferring particles in water.

Additional Characterization.

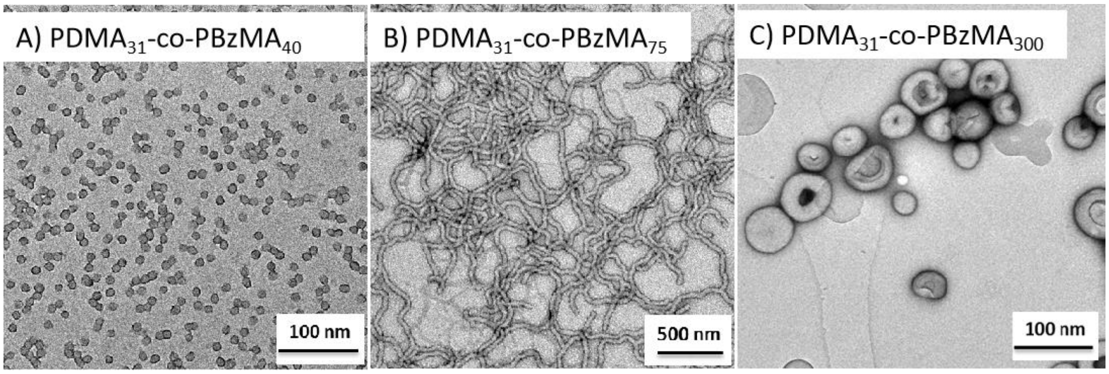

Figure S2. TEM images obtained for $PDMA_{31}$-*b*-$PBzMA_x$ diblock copolymer micelless synthesized at a total solid concentration of 20% via RAFT dispersion polymerization in ethanol. (a) Spheres (x = 40); (b) worms (x = 75) and (b) vesicles (x = 300)

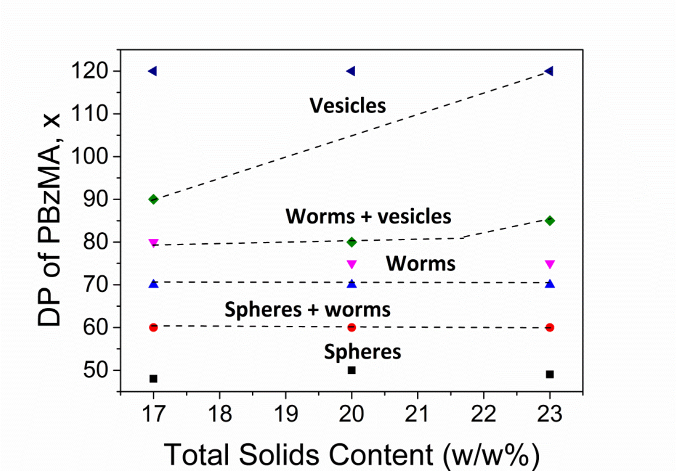

Figure S3. Phase diagram for the $PDMA_{31}$-b-$PBzMA_x$ obtained by the RAFT dispersion polymerization formulation with a systematic variation of the target degree of polymerization of PBzMA (x) block. The PISA was carried out with a PDMA macro-CTA with an average degree of polymerization of 31. The average DP of the PBzMA block in each case was determined by ^{1}H NMR spectroscopy.

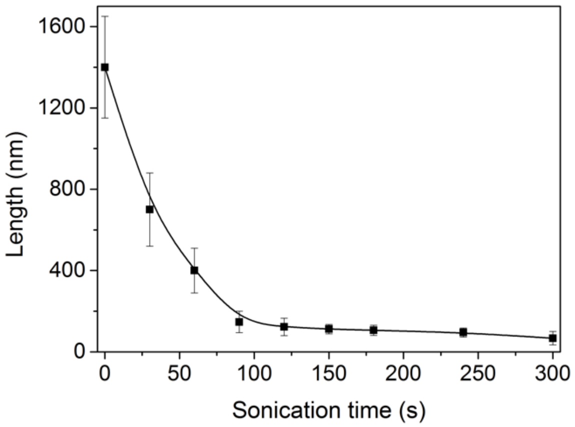

Figure S4. Effect of sonication on the length of the worm-like micelles.

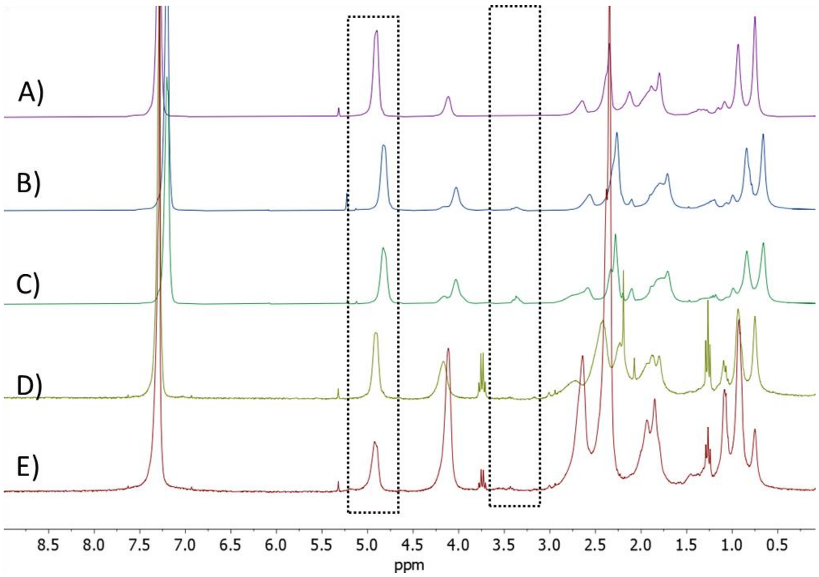

Figure S5. Crosslinking of the micelles core by the reaction between the acetoacetoxy moieties and diaminopropane. ^{1}H NMR spectra of $PDMA_{31}$-b-$PBzMA_{75}$ (A), $PDMA_{31}$-b-$PBzMA_{70}$-co-$PAAEM_{5}$ before (B) and after (C) crosslinking, and $PDMA_{31}$-b-$PBzMA_{70}$-co-$PAAEM_{10}$ before (D) and after (E) crosslinking. All the spectra were measured in $CDCl_3$.

Table S1. Quantification of the crosslinking reaction of the micelles core

Composition	NMR integration		Crosslinking density
	Peak at 4.99	Peak at 3.45	
PDMA-PBzMA	1	0.00	0%
PDMA-PBzMA-co-PAAEM$_5$ Before CL	1	0.09	-
PDMA-PBzMA-co-PAAEM$_{10}$ Before CL	1	0.18	-
PDMA-PBzMA-co-PAAEM$_5$ After CL	1	0.02	78%
PDMA-PBzMA-co-PAAEM$_{10}$ After CL	1	0.05	72%

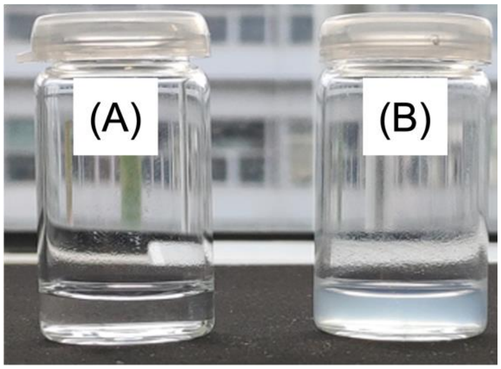

Figure S6. Photograph of the SM$_{NCC}$ A) and SM$_{HCC}$ B) after redispersion in THF

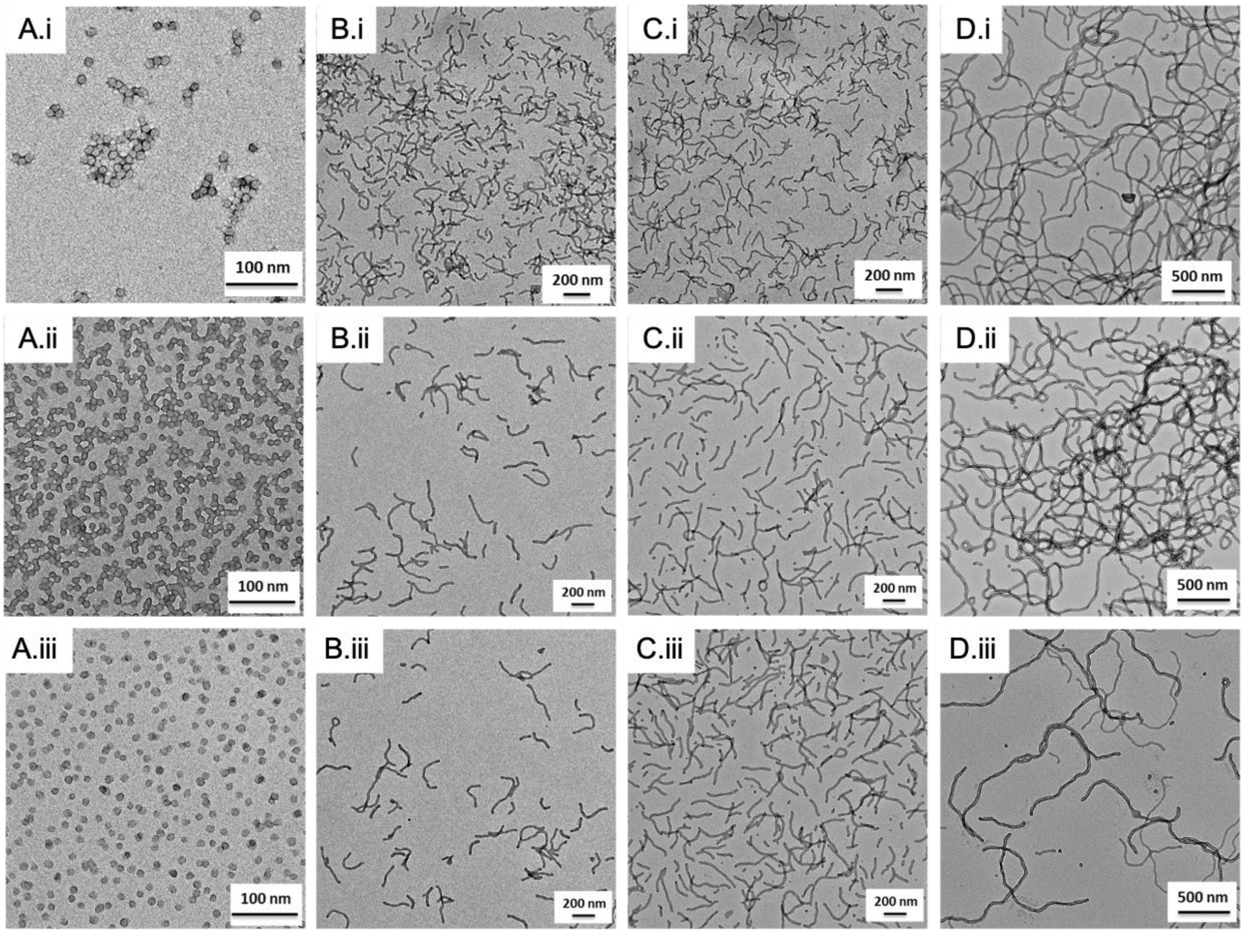

Figure S7. TEM images of the colloids studied (A) spherical colloids (SM), (B) short worm-like colloids (SWM), (C) medium worm-like colloids (MWM), (D) long worm-like colloids (LWM) with increasing crosslinking density, (i) uncrosslinked colloids (NCC), (ii) low core crosslinked colloids (LCC), and (iii) high core crosslinked (HCC).

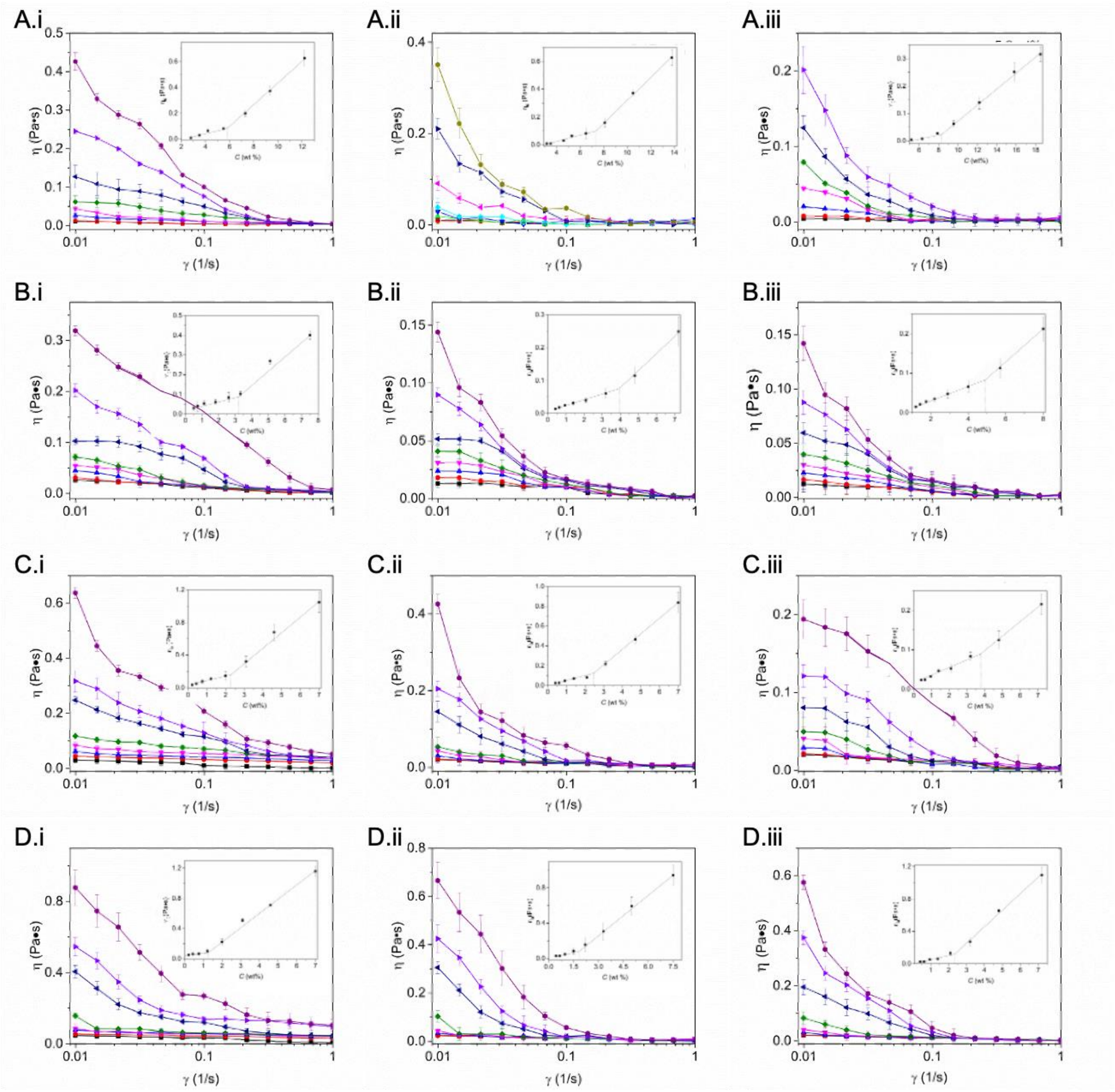

Figure S8. Dynamic viscosity of suspensions of spherical colloids (A), short worm-like colloids (B), medium worm-like colloids (C) and long worm-like colloids (D). For (i) uncrosslinked colloids (NCC), (ii) low crosslinking density colloids (LCC), and (iii) highly crosslinked colloids (HCC).

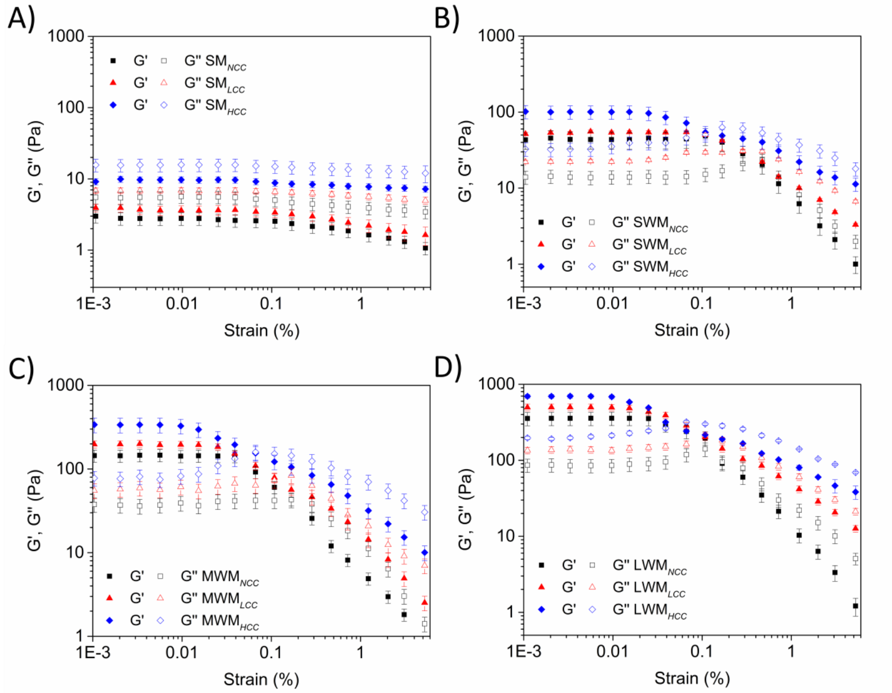

Figure S9. Strain sweep curves for colloidal dispersions (10 wt%) in water. (A) Spherical particles, (B) short worm-like particles, (C) medium worm-like particles, (D) long worm-like particles, with particles without core crosslinking (black), low core crosslinking (red), and high core crosslinking (blue).

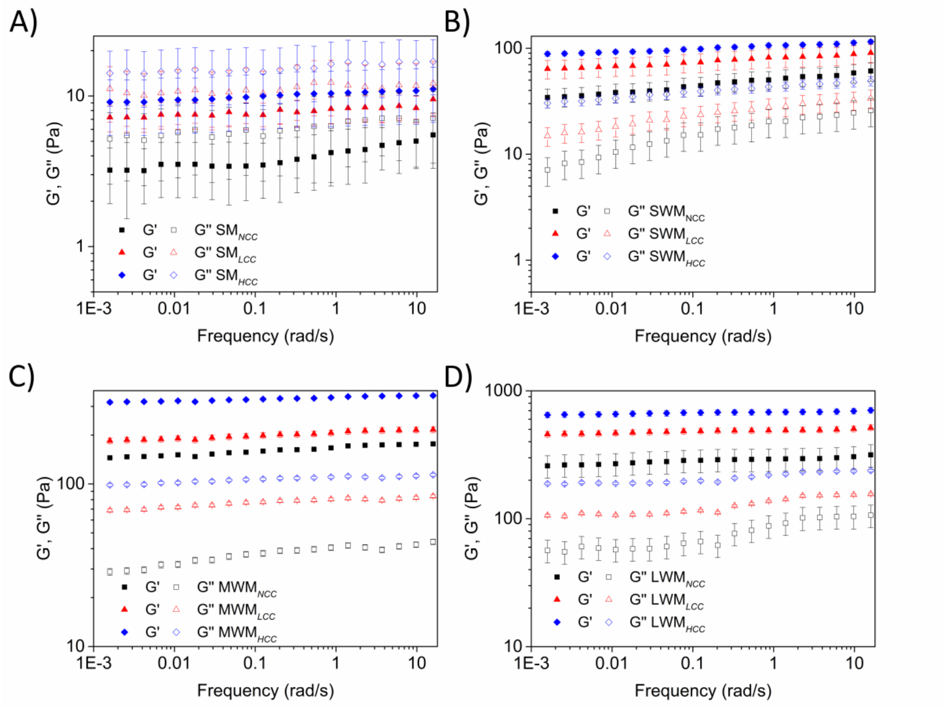

Figure S10. Frequency sweep curves for the colloidal dispersions (10wt%) in water. (A) Spherical particles, (B) short worm-like particles, (C) medium worm-like particles, (D) long worm-like particles, with particles without core crosslinking (black), low core crosslinking (red), and high core crosslinking (blue).

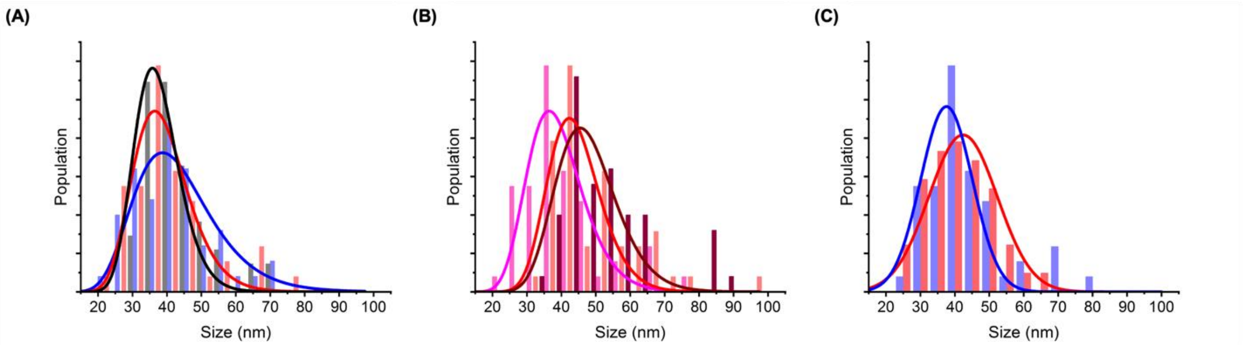

Figure S11. Width of the fibers in the gels measured from the SEM images of the gels (N>100). (A) Effect of the length of the colloids evaluated for uncrosslinked colloids. Short worm-like colloids (black), medium worm-like colloids (red), long worm-like colloids (blue). (B) Effect of the crosslinking density of the colloids core evaluated for medium worm-like colloids. Uncrosslinked colloids (NCC) (pink), moderately crosslinked colloids (LCC) (red), heavily crosslinked colloids (HCC) (burgundy). (C) Effect of the temperature of gelation of MWM_{LCC} at 25ºC (blue) and 45ºC (red).

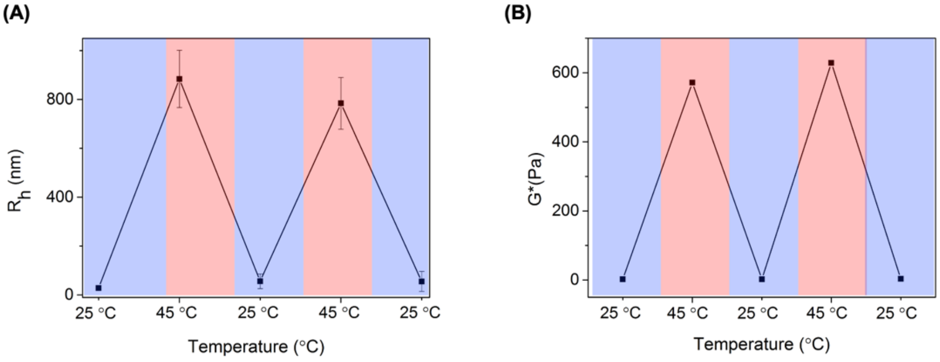

Figure S12. Reversibility of the thermally induced transition in the suspensions of spherical colloids. (A) Dynamic light scattering measurement of the size of the colloids measured in a suspension of 0.01wt% of SM_{NCC}. (B) Complex modulus of a colloidal suspension of 1wt% of SM_{NCC}.

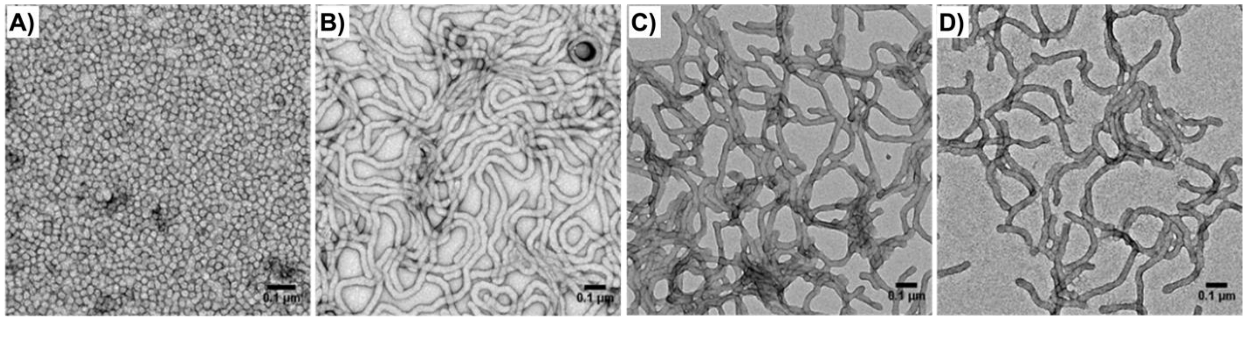

Figure S13. TEM images of micelles in water at 50 ºC. A) SM_{NCC}, B) LWM_{NCC}, C) MWM_{NCC}, D) SWM_{NCC}.

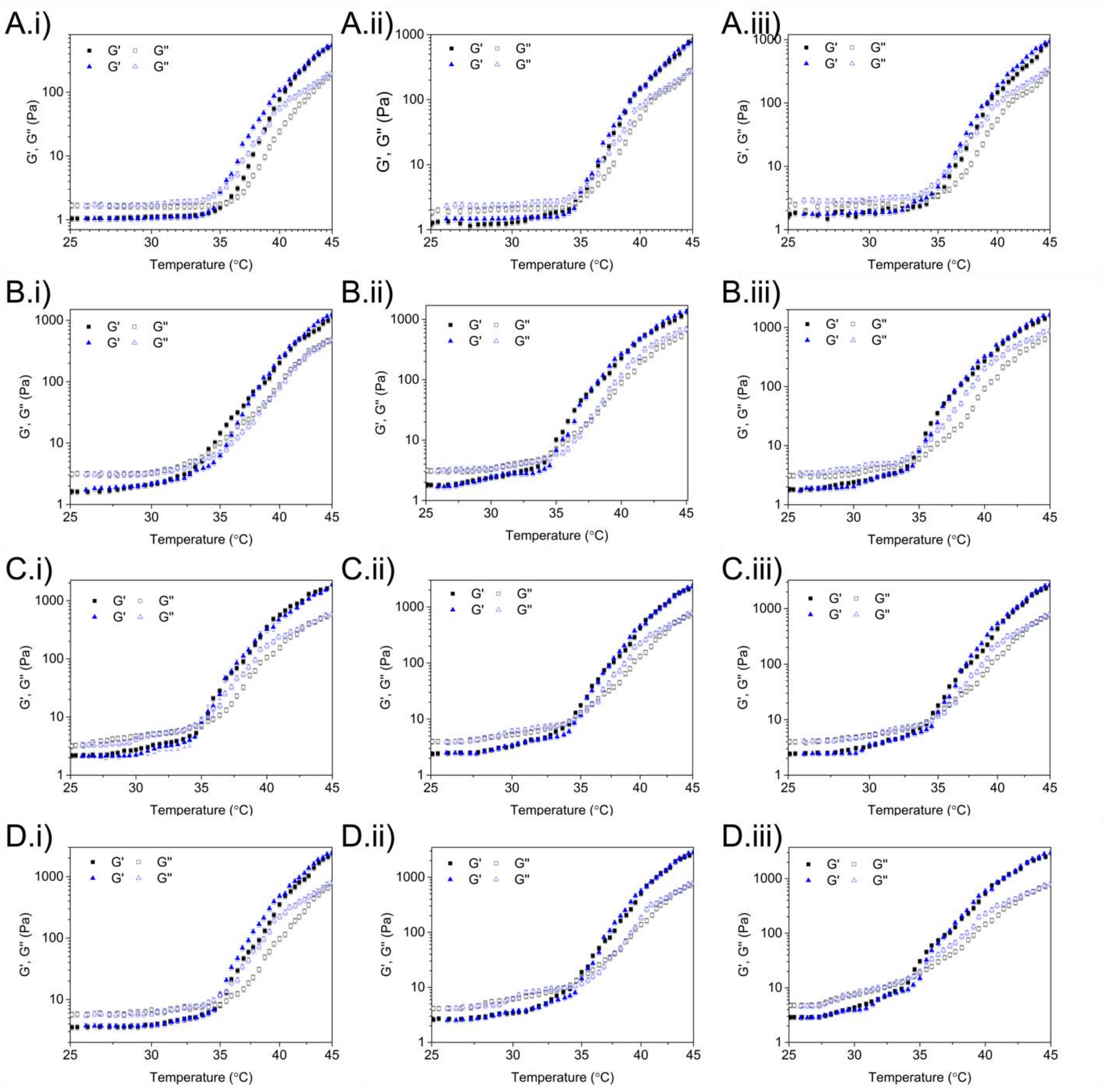

Figure S14. Variation of the storage (solid symbols) and loss (open symbols) modulus during a heating (black) cooling (blue) cycles for 1.0 wt% suspension of A) spherical colloids (SM), B) short worm-like colloids (SWM), C) medium worm-like colloids (MWM), and D) long worm-like colloids (LWM) with different crosslinking degree i) uncrosslinked colloids (NCC), ii) low core crosslinked colloids (LCC) and iii) high core crosslinked colloids (HCC).

Reference

1. E. R. Jones, M. Semsarilar, A. Blanazs, S. P. Armes, Efficient synthesis of amine-functional diblock copolymer nanoparticles via RAFT dispersion polymerization of benzyl methacrylate in alcoholic media. *Macromolecules*, **2012**, *45*, 5091-5098.
